# Receptor and secreted targets of Wnt-1/β-catenin signalling in mouse mammary epithelial cells

**DOI:** 10.1186/1471-2407-5-3

**Published:** 2005-01-10

**Authors:** Paraic A Kenny, Tariq Enver, Alan Ashworth

**Affiliations:** 1 Section of Gene Function and Regulation, Institute of Cancer Research, Fulham Rd., London, SW3 6JB, UK; 2 Breakthrough Breast Cancer Research Centre, Institute of Cancer Research, Fulham Rd, London, SW3 6JB, UK; 3 Current Address Life Sciences Division, Lawrence Berkeley National Laboratory, University of California, 1 Cyclotron Road, Mailstop 83-101, Berkeley, CA 94720, USA; 4 Current Address Molecular Haematology Unit, The Weatherall Institute of Molecular Medicine, University of Oxford, John Radcliffe Hospital, Headington, Oxford, OX3 9DS, UK

**Keywords:** wnt, catenin, microarray, hypoxia, angiogenesis

## Abstract

**Background:**

Deregulation of the Wnt/ β-catenin signal transduction pathway has been implicated in the pathogenesis of tumours in the mammary gland, colon and other tissues. Mutations in components of this pathway result in β-catenin stabilization and accumulation, and the aberrant modulation of β-catenin/TCF target genes. Such alterations in the cellular transcriptional profile are believed to underlie the pathogenesis of these cancers. We have sought to identify novel target genes of this pathway in mouse mammary epithelial cells.

**Methods:**

Gene expression microarray analysis of mouse mammary epithelial cells inducibly expressing a constitutively active mutant of β-catenin was used to identify target genes of this pathway.

**Results:**

The differential expression in response to ΔNβ-catenin for five putative target genes, Autotaxin, Extracellular Matrix Protein 1 (Ecm1), CD14, Hypoxia-inducible gene 2 (Hig2) and Receptor Activity Modifying Protein 3 (RAMP3), was independently validated by northern blotting. Each of these genes encodes either a receptor or a secreted protein, modulation of which may underlie the interactions between Wnt/β-catenin tumour cells and between the tumour and its microenvironment. One of these genes, *Hig2*, previously shown to be induced by both hypoxia and glucose deprivation in human cervical carcinoma cells, was strongly repressed upon ΔNβ-catenin induction. The predicted N-terminus of Hig2 contains a putative signal peptide suggesting it might be secreted. Consistent with this, a Hig2-EGFP fusion protein was able to enter the secretory pathway and was detected in conditioned medium. Mutation of critical residues in the putative signal sequence abolished its secretion. The expression of human *HIG2 *was examined in a panel of human tumours and was found to be significantly downregulated in kidney tumours compared to normal adjacent tissue.

**Conclusions:**

HIG2 represents a novel non-cell autonomous target of the Wnt pathway which is potentially involved in human cancer.

## Background

The Wnt/β-catenin signal transduction pathway plays a central role in metazoan development, controlling such diverse processes as cell growth, proliferation and organogenesis [1]. Wnt-1 is the prototypic member of this large family of secreted glycoproteins and was originally identified as a gene insertionally activated by mouse mammary tumour virus [2]. Wnt-1 is one of a number of Wnt family members which act to control the cellular level of β-catenin. Wnt proteins bind seven-pass transmembrane receptors of the Frizzled family, and a signal is transduced via Dishevelled to a complex which contains the Adenomatous Polyposis Coli (APC), Axin and Glycogen Synthase Kinase-3β (GSK-3β) proteins [3,4]. This signal antagonizes the phosphorylation of β-catenin by GSK-3β. There are four phosphorylation sites in the N-terminus of β-catenin which, in the absence of Wnt signal, are phosphorylated by Casein Kinase I alpha and GSK-3β [5,6]. This phosphorylation leads to the ubiquitination and subsequent proteasomal degradation of β-catenin [7]. Inhibition of β-catenin phosphorylation by Wnt signalling leads to the accumulation of β-catenin which forms a bipartite complex with members of the TCF/LEF transcription factor family and activates the transcription of target genes, a process which is regulated by multiple interacting factors [8].

Overexpression of Wnt-1 in the mammary glands of transgenic mice leads to extensive hyperplasia and tumorigenesis [9]. *APC *was identified as the tumour suppressor gene mutated in the hereditary colorectal cancer syndrome, Familial Adenomatous Polyposis [10,11]. Mutations in *Axin *and β-*catenin *have also been detected in tumours of the colon and other tissues [12]. Deregulation of this pathway appears to be play a contributory role in a significant proportion of human tumours of epithelial origin and hence, the identification of effector genes of this pathway is an important step towards the elucidation of the mechanisms involved.

Many of the Wnt targets thus far identified are cell-cycle regulators [13,14] and transcription factors [15-20], and function in a cell-autonomous manner, providing insight into the mechanisms by which tumour cells deregulate proliferation and inhibit apoptosis. Tumours are complex organs composed of tumour cells, stromal fibroblasts, endothelial cells and cells of the immune system; and reciprocal interactions between these cell types in the tumour microenvironment are necessary for tumour growth [21,22]. Here we postulate that proteins secreted by Wnt/β-catenin tumour cells and receptors expressed by these cells may play roles in mediating interactions between neighbouring tumour cells or between tumour cells and their microenvironment. Consequently, in this study we have focussed our attention on identifying novel genes encoding receptors and secreted proteins.

## Methods

### Cell culture

All reagents were purchased from Sigma unless otherwise noted. HC11 mouse mammary epithelial cells were cultured in 5% CO_2 _at 37°C in RPMI 1640, supplemented with 10% Foetal Bovine Serum, 2 mM L-glutamine, 2.5 μg/ml insulin, 5 ng/ml epidermal growth factor and 50 μg/ml gentamycin [23]. HC11-*lacZ *and HC11-Δ*N*β-*catenin *cells were routinely cultured in 2 μg/ml tetracycline to repress transcription of the tetracycline-regulated transgene. HEK293 and MDCK cells were grown in DMEM supplemented with 10% Foetal Bovine Serum.

The HC11-*lacZ *and HC11-Δ*N*β-*catenin *cell lines were generated by infecting the cells with an ecotropic retrovirus (TRE-tTA) in which the *tTA *cDNA is under the control of a tetracycline responsive promoter. Consequently, tTA expression is minimal in the presence of tetracycline and, upon tetracycline withdrawal, tTA activates its own transcription in an autoregulatory manner [24]. HC11 cells expressing tTA were subsequently infected with ecotropic retroviruses derived from RevTRE (Clontech) which directed the expression of either β-galactosidase or ΔNβ-catenin in a tetracycline dependent manner.

Bosc23 cells were used to produce ecotropic retroviruses [25]. Cells were transiently transfected with the appropriate retroviral construct and the supernatant was collected 48 hours post-transfection. Polybrene was added to a final concentration of 5 μg/ml and the supernatant was added to HC11 cells for 24 hours. HC11 cells were then subjected to antibiotic selection using either 250 μg/ml G418 or 200 μg/ml hygromycin B as appropriate.

### RNA isolation

Cell monolayers were washed twice in ice-cold Phosphate Buffered Saline and lysed by addition of Trizol (Invitrogen). Total RNA was isolated according to the manufacturer's instructions. PolyA+ RNA was purified from total RNA using Oligotex (Qiagen) according to the manufacturer's instructions.

### Northern blotting

10 μg of total RNA from each cell line was fractionated on a denaturing formaldehyde agarose gel and transferred to a positively charged nylon membrane (Hybond N+, Amersham Pharmacia Biotech) in 10x SSC. Membranes were prehybridised for four hours in 50% (v/v) formamide, 5X SSPE, 2X Denhardt's reagent, 0.1% (w/v) SDS and 100 μg/ml denatured herring sperm DNA. Radiolabelled probes were prepared from PCR-amplified cDNA clones using the Rediprime II kit (Amersham Pharmacia Biotech) according to the manufacturer's instructions. EST sequences corresponding to the coding sequence of the genes-of-interest were identified by BLAST [26] and obtained from the I.M.A.G.E. consortium through the UK Human Genome Mapping Project Resource Centre (Hinxton, UK). ESTs bearing the following I.M.A.G.E. cloneIDs were used: *Autotaxin *– 533819; *CD14 *– 2936787; *Ecm1 *717050; *Hig2 *– 367488; *Ramp3 *– 615797, *HIG2 *4366895). Following overnight hybridisation with the labelled probe, the membranes were washed twice in 1X SSC, 0.1% (w/v) SDS at room temperature for 20 mins, and twice in 0.2X SSC, 0.1% (w/v) SDS at 68°C for 10 mins and exposed to film at -80°C for 48 hours. Bound probe was quantitated using a phosphorimager (Molecular Dynamics).

### Western blotting

Cell monolayers were rinsed twice with ice-cold Phosphate Buffered Saline and total cell lysates were prepared by scraping cells into a minimal volume of 50 mM Tris. HCl pH 7.5, 150 mM NaCl, 0.5% NP40 and Complete protease inhibitor cocktail (Roche). Aliquots containing 80 μg protein from each sample were analysed by SDS-PAGE [27], and transferred electrophoretically to a PVDF membrane. Mouse monoclonal antibodies were used to detect tTA (Clontech), β-catenin (Transduction Laboratories) and EGFP (Santa Cruz Biotechnology). Samples of conditioned medium were concentrated 12-fold using Microcon YM-10 centrifugal filter units (Millipore) prior to analysis.

### Construction of plasmids

A BgIII fragment containing the *lacZ *cDNA was excised from the CMV-*lacZ *construct (a gift of Trevor Dale) and sub-cloned into BamHI digested RevTRE to make RevTRE-*lacZ*. A plasmid containing a myc-tagged ΔNβ-catenin was obtained from Hans Clevers. The myc-tagged ΔNβ-catenin was excised with KpnI and NotI and the ends were blunted, and subcloned into HpaI digested RevTRE to make RevTRE-Δ*N*β-*catenin*. The mouse *Hig2 *open reading frame was amplified by PCR from I.M.A.G.E. cDNA clone 367488 using the primers TTTACTAGTAGGAGCTGGGCACCGTCGCC and TTTTACCGGTGCCTGCACTCCTCGGGATGGATGG. The PCR product was digested with AgeI and SpeI and subcloned into the AgeI and NheI sites in pEGFP-C1 (Clontech) to make the Hig2-EGFP fusion gene. Site directed mutagenesis was carried out by the method of Sawano and Miyawaki (2000) [28]. The primer TGCTGAACCTCGAGGAGCTGGGCATCATG was used to make the Hig2-EGFP(Y8V9/D8D9) mutant.

### Transient transfections

Transient transfections were performed using Lipofectamine (Invitrogen) according to the manufacturer's instructions. Briefly, 1.5 × 10^5 ^cells were plated in 3.5 cm wells on the day prior to transfection. Each well was transfected with a total of 0.9 μg DNA under serum-free conditions for six hours, after which the cells were washed and incubated for a further 48 hours before assaying expression.

### β-galactosidase activity assay

For the tetracycline dose response curve, 5000 HC11-*lacZ *cells for each condition, were cultured in triplicate in 96 well plates for 72 hours, and beta-galactosidase activity was determined as previously described [24].

## Results

### Generation of HC11-*lacZ *and HC11-Δ*N*β-*catenin *cell lines

Stable cell lines were generated in which either ΔNβ-catenin or β-galactosidase was expressed in a tetracycline dependent manner. These cell lines were established using a novel autoregulatory system in which the expression level of the tetracycline transactivator (tTA) protein is minimised during routine culture and is induced upon withdrawal of tetracycline with concomitant upregulation of the transgene-of-interest [24]. This strategy helps to minimise deleterious effects due to tTA toxicity.

**Figure 1:**
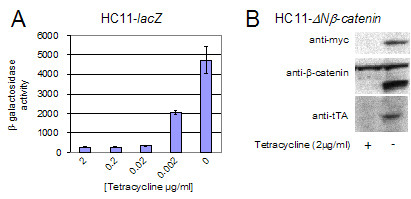
Generation of stable HC11 cell lines expressing β-galactosidase and Δ Nβ-catenin in a tetracycline dependent manner. **(a) **HC11-*lacZ *cells – β-galactosidase activity of cell lysates following incubation for three days at the indicated tetracycline concentrations. β-galactosidase expression is effectively repressed at 20 ng/ml tetracycline. The assay was carried out in triplicate and results are presented as the mean β-galactosidase activity (normalised to the protein concentration of the samples) ± standard error. **(b) **Western blot of total cell lysates from HC11-Δ*N*β-*catenin *cells cultured ± tetracycline. The N-terminal deletion mutant of β-catenin is detected by both its myc-epitope tag and the anti-β-catenin antibody. tTA was detected only in the absence of tetracycline, confirming the autoregulatory nature of the induction system.

A dose-response analysis for the HC11-*lacZ *cell line is shown in Figure 1A. β-galactosidase expression is effectively repressed at tetracycline concentrations in excess of 20 ng/ml and is strongly induced in the absence of tetracycline. The N-terminal truncation mutant of β-catenin can be detected by western blotting by both its myc-epitope tag and an anti-β-catenin antibody (Figure 1B). tTA expression is detectable only in the absence of tetracycline demonstrating the autoregulatory nature of this system.

### Microarray analysis

Transgene expression was induced in HC11-*lacZ *and HC11-Δ*N*β-*catenin *cells by withdrawal of tetracycline for 72 hours. Total RNA was isolated, from which mRNA was purified. cDNAs were labelled and hybridized to an 8962 element Incyte mouse GEM1 cDNA microarray (Incyte Genomics, Palo Alto, CA). These data are provided as supplementary material (See Additional file 1). Among those genes upregulated were two genes shown by other workers to be transcriptional targets of this pathway – *Fibronectin *[29] and *Autotaxin *[30] (data not shown) – suggesting that our model of Wnt/β-catenin signalling deregulation results in the activation of a set of target genes which overlaps, at least partially, with pathway targets in other cell lines. The microarray experiment described here was performed only once but differential expression was repeatedly validated by northern blotting from independent samples for the genes discussed here.

### Validation of targets

**Figure 2:**
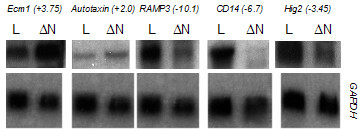
Confirmation of differential expression of putative target genes in response to ΔNβ-catenin overexpression. The expression level of each gene in the HC11-*lacZ *(L) and HC11-Δ*N*β-*catenin *(ΔN) cell lines was assessed by Northern blotting. The fold-induction was quantitated using a phosphorimager under non-saturating conditions, normalised to the expression of GAPDH mRNA in the samples and is expressed as fold change relative to the expression level of that gene in the HC11-*lacZ *cell line.

Five genes were selected for further study – *Extracellular Matrix Protein 1 *(*Ecm1*), *Autotaxin*, *Receptor Activity Modifying Protein 3 *(*Ramp3*), *Cd14 *and *Hypoxia Inducible Gene 2 *(*Hig2*). Each putative target gene was initially subjected to a secondary screen by Northern blotting to confirm the differential expression in response to ΔNβ-catenin (Figure 2). RNA samples used for Northern blotting were from independent induction experiments to those used for microarray analysis, thus demonstrating repeatedly by two distinct methods that the transcript levels of these genes are altered in cells overexpressing ΔNβ-catenin. The expression level of each of the transcripts was quantitated using a phosphorimager and normalised to the expression of *Gapdh *mRNA in the samples. The data in Figure 2 represent film exposure times ranging between 24 and 72 hours. Quantitations were performed using short (one hour or less) exposures to a phosphorimager screen, such that the signal intensity was not saturating.

### Molecular cloning of mouse *Hig2*

**Figure 3:**
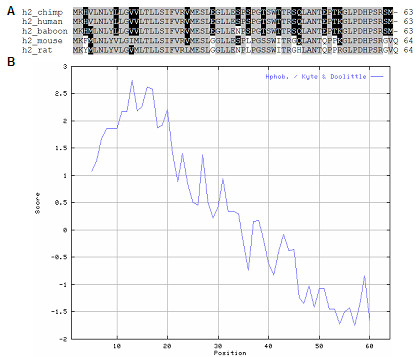
Sequence analysis of five mammalian Hig-2 proteins. **(a) **Multiple sequence alignment of Hig2 amino acid sequences of mouse, rat, human, chimpanzee and olive baboon shows that these proteins share strong sequence similarity. **(b) **A representative Kyte-Doolittle hydrophobicity plot using the mouse Hig2 amino acid sequence indicates these proteins have hydrophobic N-termini, reminiscent of a signal peptide.

*Hypoxia Inducible Gene 2 *encodes a 63 amino acid polypeptide and was one of several genes identified in a screen for genes regulated by hypoxia in a human cervical epithelial cell line [31]. HIG2 shares no sequence similarity with other known proteins. In order to facilitate the functional analysis of this gene, ESTs were identified which encoded mouse and rat Hig2, and the sequences of chimpanzee and baboon were inferred from genomic sequence data. A multiple alignment of the inferred amino acid sequences shows that these polypeptides are highly similar (Fig 3A). Analysis of these sequences using a Kyte-Doolittle hydrophobicity plot showed that the N-termini of these proteins contain a series of hydrophobic amino acids (Fig 3B). This region of hydrophobicity was reminiscent of a signal peptide and sequence analysis using the signal peptide prediction program, SignalP [32,33] supported this possibility.

### Hig2 has an N-terminal signal peptide and is secreted

**Figure 4:**
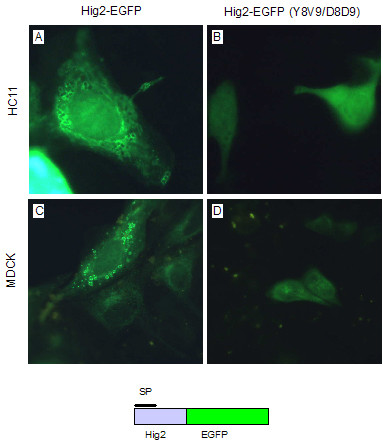
Subcellular localisation of a Hig2-EGFP fusion protein and mutational analysis of the putative signal peptide in HC11 and MDCK cells. **(a **and **c) **Hig2-EGFP is localised to large spherical vesicles located in the cytoplasm. **(b **and **d) **The introduction of two aspartate residues into the putative hydrophobic signal peptide abolishes the vesicular subcellular localisation of the mutant protein.

**Figure 5:**
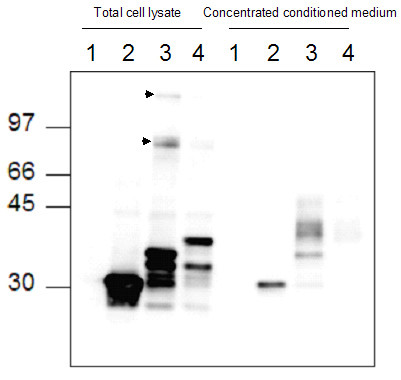
Hig2-EGFP fusion protein is secreted from transfected cells. Western blot analysis of total cell lysates and conditioned medium from (1) untransfected HEK293 cells and cells transfected with plasmids encoding (2) EGFP, (3) Hig2-EGFP, (4) Hig2(Y8V9/D8D9)EGFP. Oligomers of HIG2-EGFP formed by intermolecular disulphide bridges are indicated by arrowheads.

To investigate the subcellular localisation of Hig2, a *Hig2*-*EGFP *fusion gene was constructed and expressed in both HC11 and Madin-Darby Canine Kidney (MDCK) cells by transient transfection (Figure 4a and 4c). In both cell lines, Hig2-EGFP is localised to large round vesicle-like structures in the cytoplasm. Similar observations were made in HEK-293 cells (data not shown). The fluorescence was detected predominantly around the periphery of these structures suggesting that they do not consist of solid masses of aggregated protein. When two aspartate residues were introduced to the putative signal peptide by site-directed mutagenesis, Hig2(Y8V9/D8D9)EGFP, this distinctive subcellular localization was abolished (Fig 4B and 4D). These large structures did not colocalize with either markers of mitochondria (pDsRed2-mito, Clontech), nor lipid droplets (Nile Red, Molecular Probes) nor with markers of endosomes or lysosomes (pulse-chase analysis with TRITC-dextran); data not shown. However, in live HC11 cells transfected with Hig2-EGFP, observations at high magnification revealed that the cytoplasm of these cells contained many very small solid green vesicles moving along the cytoskeleton. These vesicles were approx 1/100 the size of the large vesicles shown in Fig 4A and 4C, and were not observed in cells transfected with either Hig2(Y8V9/D8D9)EGFP or EGFP alone. The rapidity of this motion in live cells, even at room temperature, precluded capture of these images but suggested the possibility that measurable amounts of secreted Hig2-EGFP might be found in the culture medium. HEK-293 cells were chosen as they could be transfected at high efficiency (approx 80%), the presence of the green transport vesicles was confirmed, and 48 hours after transfection samples of total cell lysate and conditioned medium were analysed by western blotting. Secreted Hig2-EGFP was detected in the conditioned medium of Hig2-EGFP cells, but not Hig2(Y8V9/D8D9)EGFP cells (Fig 5). Multiple bands were detected in cell lysates for both Hig2-EGFP fusion proteins: whether these represent artifactual degradation products or physiologically relevant biological entities is as yet unknown. Such multiple banding has also been observed with other EGFP-fusion proteins targeted to the secretory pathway (Amphiregulin-EGFP, PK unpublished observations). At least one of the bands may result from internal translation initiation at the consensus Kozak initiation sequence of pEGFP-C1 which is located between the Hig2 and EGFP open reading frames.

EGFP was also detected in the conditioned medium. This is consistent with previous reports of GFP secretion via a non-classical Brefeldin A-insensitive pathway [34]. In this study, several cell lines are described (including HEK293) in which wild-type GFP is released from the cell without passing through the golgi apparatus. Thus, it is formally possible that, instead of its secretion being directed by the putative signal peptide, HIG2-EGFP might be released from the cell via this pathway in a manner specifically dependent on the EGFP moiety. The presence of post-translational modifications acquired during endoplasmic reticulum/golgi apparatus mediated secretion would exclude the latter hypothesis. The altered mobility of HIG-2-EGFP in the medium suggested that it might be glycosylated, however the mobility was not changed by treatment with the glycosidase PNGaseF, suggesting that this secreted protein is not glycosylated (data not shown). Previous studies using GFP fused to a signal peptide directing entry into the ER demonstrated that, in this redox environment, the cysteine residues of GFP form intermolecular disulphide bridges which result in oligomerization of GFP molecules [35]. Oligomers of Hig2-EGFP were detected (Fig 5, black arrowheads) but no oligomerization of EGFP was observed. Hig2 itself does not contain cysteine residues, thus the oligomerization is mediated by the EGFP domains. These data are consistent with HIG2-EGFP entry into the classical secretory pathway. Collectively, these data demonstrate that Hig2 contains a functional N-terminal signal peptide and is likely a secreted protein.

### Expression of *HIG2 *in human tumours

**Figure 6:**
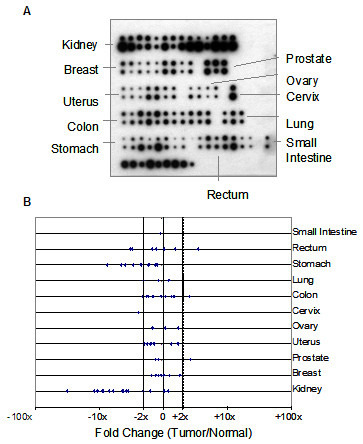
**(a) **Analysis of *HIG2 *mRNA expression on a human cDNA array containing matched tumour and normal samples from 68 patients. For each pair, the tumour sample is on top. The bottom row of the array contains cDNA from cell lines (left to right: HeLa, Daudi, K562, HL-60, G361, A549, MOLT-4, SW480 and Raji). **(b) **Quantitative analysis by phosphorimager. Vertical dashed lines indicate ± 2-fold difference. *HIG2 *expression is broadly similar in most pairs, but is strongly and consistently downregulated in most of the kidney and stomach tumours analysed.

To investigate the relevance of *HIG2 *in human tumours, the expression level of this gene was examined in 68 tumour cDNA samples compared to normal adjacent tissue from the same patients using a Matched Tumour/Normal cDNA blot (Clontech) (Figure 6A). The levels of *HIG2 *were approximately similar in most of the tumour types examined but were strongly and consistently downregulated in most of the cases of kidney and stomach tumours analysed. These data suggest that the downregulation of *HIG2 *observed upon deregulated β-catenin signalling *in vitro *may be of clinical relevance in human tumours.

## Discussion

cDNA microarray analysis of the transcriptional changes resulting from overexpression of a constitutively active β-catenin revealed a panel of putative target genes of the Wnt/β-catenin pathway in mouse mammary epithelial cells. This differential expression was confirmed by Northern blotting in five cases.

Autotaxin was originally identified as a secreted enzyme with potent motility stimulating activity [36] and has both pyrophosphatase and phosphodiesterase activity [37]. Transplantation experiments in athymic mice showed that ras-transformed NIH-3T3 fibroblasts became significantly more tumorigenic, invasive and metastatic when transfected with *Autotaxin *[38], and purified recombinant Autotaxin has potent angiogenic activity *in vivo *[39]. Autotaxin has been shown to be regulated by both Wnt-1 and retinoic acid [30]. Autotaxin has been shown to have lysophospholipase activity and the effects of Autotaxin on tumour cell motility are mediated by its conversion of lysophosphatidylcholine to lysophosphatidic acid (LPA), a potent signalling molecule [40,41].

Extracellular Matrix Protein 1 was first identified as a novel 85 KDa protein secreted by a mouse osteogenic stromal cell line [42]. *In situ *hybridisation showed that *Ecm1 *was strongly expressed in most newly formed blood vessels and experiments using purified recombinant Ecm1 showed that it could increase the proliferation rate of vascular endothelial cells *in vitro *and also stimulate angiogenesis *in vivo*. The ability to induce *de novo *angiogenesis is an absolute requirement for tumours to grow beyond a size which can be readily perfused by oxygen and nutrients from the interstitial fluid. ECM1 is overexpressed in many epithelial tumours including 73% of breast tumours analyzed [43]. Homozygous loss-of-function mutations in the human ECM1 gene were recently identified by linkage analysis as the causative mutations behind Lipoid Proteinosis, a rare autosomal recessive disorder characterized by hyaline deposition in the skin, mucosae and viscera [44]. The identification of *Autotaxin *and *Ecm1 *as genes upregulated by activation of this pathway, together with VEGF [45] suggests that deregulation of Wnt/β-catenin signalling during tumour initiation and progression may be one of the factors which promotes tumour angiogenesis.

*CD14*, which can function as both a receptor and a secreted protein, was downregulated upon ΔNβ-catenin expression. CD14 is a glycosyl-phosphatidylinositol-linked cell surface protein, preferentially expressed in monocytes, where it acts as a receptor for Lipopolysaccharide Binding Protein:Lipopolysaccharide complexes [46]. Soluble CD14 (sCD14) is also expressed in mammary epithelial cells *in vitro *and has been detected in human milk where it is postulated to play a role in neonatal immunity [47], and is strongly upregulated in mammary luminal epithelial cells *in vivo *at the onset of involution [48].

*Receptor Activity Modifying Protein 3 *(*RAMP3*) was downregulated upon ΔNβ-catenin induction and is one of three members of the RAMP family. These proteins are involved in mediating the cellular response to the neuropeptides calcitonin, calcitonin gene related peptide, amylin and adrenomedullin. The RAMP family members function as chaperones for the seven transmembrane domain G-protein coupled receptors for these neuropeptides, shuttling the receptor to the cell surface and altering receptor glycosylation. The ligand binding phenotype of the receptor is dependent on the RAMP family member with which it is associated [49]. RAMP3-Calcitonin Receptor (CR) heterodimers form a functional receptor for amylin [50], and RAMP3-Calcitonin-Receptor-Like-Receptor (CRLR) heterodimers act as an adrenomedullin receptor [51].

Expression of both CR and CRLR was detected in HC11 by RT-PCR (data not shown) suggesting that functional receptor-RAMP complexes are present in this cell line. Adrenomedullin, the ligand for the CRLR/RAMP3 receptor dimer, functions as a growth factor in several human tumour cell lines [52], in addition to promoting angiogenesis *in vivo *[53] via CRLR/RAMP3 and CRLR/RAMP2 receptor dimers [54,55].

*Hypoxia*-*inducible gene 2 *(*Hig2*) was one of several genes identified in a representational difference analysis screen for genes regulated by hypoxia in a human cervical epithelial cell line. The human gene encodes a 63 amino acid polypeptide of unknown function [31]. Expression of mouse *Hig2 *was downregulated in HC11 cells overexpressing ΔNβ-catenin. The identification of a group of mammalian orthologues revealed a well conserved hydrophobic region in the N-terminus, reminiscent of a signal peptide. A Hig2-EGFP fusion protein entered the secretory pathway and was detected in conditioned medium of transfected cells. The introduction of a pair of charged amino acids into the hydrophobic region abolished secretion, lending support to the hypothesis that this region contains a functional signal peptide. The nature of the large vesicular structures observed in Hig2-EGFP overexpressing cells is as yet unclear. Mammary epithelial cells are known to contain membrane-enclosed lipid droplets, as well as a variety of vesicular compartments involved in the secretion of casein, citrate, lactose and calcium [56], however the presence of these vesicles in MDCK and HEK293 cells argues that they are not mammary specific. Indeed, co-localization experiments suggest that these structures are neither mitochondria, lysosomes, endosomes nor lipid droplets. Given the demonstration that Hig2 is secreted, these structure most likely correspond to overexpressed Hig2-EGFP in transit through the endoplasmic reticulum and golgi apparatus. As no antibody is available against Hig2, it was not possible to investigate the localisation of the endogenous protein, but these data represent a useful initial step in the functional characterisation of this gene.

Analysis of the expression of *HIG2 *using a matched Tumour/Normal tissue cDNA array showed that *HIG2 *is widely expressed. In most cases, the levels of *HIG2 *in the tumours and the associated normal tissue controls were similar. *HIG2 *was, however, strongly and consistently downregulated in the majority of the kidney and stomach tumours analysed. This represents an significant validation of our *in vitro *findings in human tumours, and suggests that HIG2 may exert a tumour suppressive effect *in vivo*. Human HIG2 is located on 7q32.2, a commonly deleted region in several tumour types, most prominently leukaemias and lymphomas [57]. Deletion analysis of 7q in a panel of patients with Splenic Lymphoma with Villous Lymphocytes by Catovsky and colleagues suggests that a critical tumour suppressor is located on 7q32 [58].

## Conclusions

The identification of this panel of candidate target genes for this clinically important signal transduction pathway adds to those identified by other workers in a variety of model systems and suggests that, as well as promoting tumour cell proliferation and survival in a cell autonomous manner, this activation of this pathway is likely to have a series of non-cell autonomous effects. Here we have focussed on the identification of Wnt/β-catenin target genes that are either secreted signalling molecules or receptors. It is likely that such targets are involved in mediating autocrine proliferation, promotion of angiogenesis and the mediation of reciprocal communication between Wnt/β-catenin tumours and their microenvironmental milieu.

## Competing interests

The authors declare that they have no competing interests.

## Authors' contributions

PK carried out all of the experimental procedures and drafted the manuscript. PK, TE and AA contributed to the design of the study. All authors read and approved the final version of this manuscript.

## Pre-publication history

The pre-publication history for this paper can be accessed here:



## Supplementary Material

Additional File 1 cDNA microarray dataset: HC11 ΔNβ-catenin v. HC11 lacZ. This file contains the dataset from the Incyte GEM1 cDNA microarray comparison between HC11 cells overexpressing ΔNβ-catenin (Probe 1) and β-galactosidase (Probe 2). This file may be easily imported into MS Excel, Genespring or other microarray analysis software to facilitate further analysis. Click here for file
